# Cardiac defects of hypermobile Ehlers-Danlos syndrome and hypermobility spectrum disorders: a retrospective cohort study

**DOI:** 10.3389/fcvm.2024.1332508

**Published:** 2024-03-18

**Authors:** Dacre R. T. Knight, Katelyn A. Bruno, Ayush Singh, Bala Munipalli, Shilpa Gajarawala, Mahima Solomon, S. Christian Kocsis, Ashley A. Darakjian, Angita Jain, Emily R. Whelan, Archana Kotha, David J. Gorelov, Sabrina D. Phillips, DeLisa Fairweather

**Affiliations:** ^1^ Department of General Internal Medicine, Mayo Clinic, Jacksonville, FL, United States; ^2^ Department of Cardiovascular Medicine, Mayo Clinic, Jacksonville, FL, United States; ^3^ Division of Cardiovascular Medicine, Department of Medicine, University of Florida, Gainesville, FL, United States; ^4^ Center for Clinical and Translational Science, Mayo Clinic, Rochester, MN, United States; ^5^ Mayo Clinic Graduate School of Biomedical Sciences, Mayo Clinic, Rochester, MN, United States; ^6^ Department of Immunology, Mayo Clinic, Jacksonville, FL, United States

**Keywords:** echocardiogram, aortic root dilation, hypermobility, valve prolapse, prevalence

## Abstract

**Background:**

Defective connective tissue structure may cause individuals with hypermobile Ehlers-Danlos syndrome (hEDS) or hypermobility spectrum disorders (HSD) to develop cardiac defects.

**Methods:**

We conducted a retrospective chart review of adult patients treated in the EDS Clinic from November 1, 2019, to June 20, 2022 to identify those with cardiac defects. Echocardiogram data were collected using a data collection service. All EDS Clinic patients were evaluated by a single physician and diagnosed according to the 2017 EDS diagnostic criteria. Patient demographic, family and cardiac history were extracted from self-reported responses from a REDCap clinical intake questionnaire. Patients with at least 1 available echocardiogram (ECHO) were selected for the study (*n *= 568).

**Results:**

The prevalence of aortic root dilation in patients with hEDS was 2.7% and for HSD was 0.6%, with larger measurements for males than females and with age. Based on self-reported cardiac history that was verified from the medical record, patients with hEDS with bradycardia (*p* = 0.034) or brain aneurysm (*p* = 0.015) had a significantly larger average adult aortic root z-score. In contrast, patients with HSD that self-reported dysautonomia (*p* = 0.019) had a significantly larger average aortic root z-score. The prevalence of diagnosed mitral valve prolapse in patients with hEDS was 3.5% and HSD was 1.8%. Variants of uncertain significance were identified in 16 of 84 patients that received genetic testing based on family history.

**Conclusions:**

These data reveal a low prevalence of cardiac defects in a large cohort of well-characterized hEDS and HSD patients. Differences in cardiovascular issues were not observed between patients with hEDS vs. HSD; and our findings suggest that cardiac defects in patients with hEDS or HSD are similar to the general population.

## 1 Introduction

Hypermobile Ehlers-Danlos syndrome (hEDS) and hypermobility spectrum disorders (HSD) are common connective tissue disorders that have a strong genetic basis from family history yet no identified genetic variant/s (1, 2). Other forms of EDS such as vascular EDS are associated with joint hypermobility and serious cardiovascular complications including arterial aneurysms, dissections, and organ rupture (3, 4). In contrast, mild, nonprogressive aortic root dilatation (ARD) and mitral valve prolapse (MVP) have been associated with hEDS and HSD, which have been reported to occur in 6%–21% of patients with hEDS/HSD (5–7). In 2017 new diagnostic criteria for hypermobile EDS were created that separated patients into hEDS or HSD categories. Although the presence of ARD and MVP are part of the diagnostic criteria distinguishing hEDS from HSD, their presence is not required for a diagnosis of hEDS (2). Based on previous reports that ARD and MVP were present more often in patients with symptomatic hypermobility than the general population, the 2017 diagnostic criteria indicated an echocardiographic assessment for cardiovascular complications (2). As a result, echocardiography is often a routine part of the evaluation of joint hypermobility syndromes.

However, recent studies have produced contradictory findings. In a study published in 2017, Ritter et al. examined mainly pediatric patients diagnosed with hEDS age 7–21 and reported ARD in 14.2% (Z-score >2.0) or 5.5% (Z-score >3.0) of patients (6). Asher et al. combined their findings from adult patients diagnosed with hEDS, HSD and classical EDS and found ARD in 1.6% and MVP in 6.4% of patients (5). A study by Paige et al. that examined 95 pediatric patients diagnosed with cEDS (*n* = 12) or hEDS (*n* = 83) using the 2017 diagnostic criteria found only one patient with mild ARV and another with mild ascending aorta dilation (8). Recently, Rashed et al. examined 258 mainly adult patients (94%) diagnosed with hEDS or HSD using the 2017 diagnostic criteria at their specialty Cardiovascular Genetics Program and reported 15.2% ARD and 7.5% MVP in hEDS/HSD combined (7). They also reported that ARD occurs more often in patients diagnosed with hEDS (20.7%) than with HSD (7.7%) and occurs at a similar prevalence between females and males, although was mild in >90% of females and moderate-to-severe in 50% of males (7). They also observed cervical artery dissection (CeAD, *n *= 2), spontaneous coronary artery dissection (SCAD, *n *= 2), and SCAD plus celiac artery pseudoaneurysm (*n* = 1) in several patients (1.9%). We established a specialty EDS Clinic in the fall of 2019 (9). Based on previous studies, we hypothesized that hEDS and HSD patients diagnosed in our clinic would have a higher rate of abnormal echocardiogram (ECHO) findings than the general population. In this study we retrospectively investigated a large cohort of adult patients diagnosed with hEDS or HSD (*n *= 568) using the 2017 diagnostic criteria in order to determine the prevalence of patients with ARD, MVP or other cardiac complications and to determine whether cardiac defects or comorbidities differed between patients with hEDS or HSD.

## 2 Methods

### 2.1 Ethics statement

This retrospective study was approved by the Mayo Clinic Institutional Review Board (IRB# 19-011260) and informed consent was waived by the IRB for all patients. The research conformed to the principles outlined in the Declaration of Helsinki.

### 2.2 Study design

All patients seen at the EDS Clinic were evaluated by the same physician who is an EDS specialist and diagnosed according to the 2017 EDS diagnostic criteria (2). Adult patients received a diagnosis of hEDS or HSD, which we reported previously have a high overlap of symptoms and comorbidities (10). Patients that attended the EDS Clinic but did not obtain a diagnosis of hEDS or HSD were also examined. These patients are not healthy, but have many symptoms and comorbidities similar to hEDS and HSD patients. It is possible that some of these patients have symptomatic hypermobility for joints other than those assessed by the Beighton score or were stiffer as occurs with age and so may not have met the diagnostic criteria. A full medical history and physical exam were obtained at the EDS Clinic visit. A retrospective chart review of patients seen at the EDS Clinic at Mayo Clinic in Jacksonville, Florida was conducted from November 1, 2019, to June 20, 2022. From the total cohort of 999 patients, 233 patients were diagnosed with hEDS (met criteria 1, 2 and 3) with 170 having at least one echocardiogram (ECHO) and 148 with an aortic root measurement. Additionally, 551 patients were diagnosed with HSD (met criteria 1 and 3 but NOT 2) with 398 having at least one ECHO and 333 with an aortic root measurement. From all individuals visiting the EDS Clinic during that time window, 8 had another connective tissue disorder (did not meet criteria 3) and were not included in the study, and 158 did not meet criteria 1, 2, or 3 with 101 of these patients having at least one ECHO and 77 with an aortic root measurement. For multiple reasons, data were not reported for 49 patients for a total of 784 patients in the study. Only 568 of the patients had at least one ECHO in their medical record and were used in the study.

### 2.3 Patient REDCap intake questionnaire

Self-reported demographics, patient history and cardiac history were obtained from all patients who attended the EDS Clinic using a Research Electronic Data Capture (REDCap) Intake Questionnaire, as reported previously (10).

### 2.4 Echocardiography measurements

Echocardiogram data were collected retrospectively using the cardiovascular DataMart; a data collection service offered by the Mayo Clinic that extracts data from the electronic medical record (EMR). Echocardiogram data available in the EMR for patients seen at the EDS Clinic was included in the study, including echocardiograms that were obtained prior to the patient being seen at the EDS Clinic. This was a retrospective study and so no active followup occurred.

The aortic root diameter was measured at end-diastole at the onset of the QRS from the leading edge of the aortic root from the parasternal long axis image on transthoracic echocardiography. See the Supplementary Excel File for the echocardiography data dictionary that describes how the specific measurements were obtained. To understand the cardiac complications of aortic root dilation (ARD), aortic root z-scores for adults were calculated by the study team (not DataMart) using the Devereux formula (11). This involved considering the aortic root diameter and patient age while accounting for weight and height through body surface area (BSA) with the calculation below, following recent recommendations by (12, 13):

$$\mathrm{BSA}=0.007184\times (\mathrm{height}\;(\mathrm{cm}{)}^{0.725})\times (\mathrm{weight}\;(\mathrm{kg}{)}^{0.425})$$

A z-score greater than 2 was considered dilated. A diagnosis of MVP and/or mitral valve regurgitation (MVR) was confirmed by an echocardiologist that was an expert in these analyses which were conducted in the Department of Cardiovascular Medicine at Mayo Clinic.

### 2.5 Genetic testing

A patient with a diagnosis of hEDS or HSD is not indicated for genetic testing because there is no known gene with a causal relationship for these diagnoses. However, some clinical features or medical/ family history indicate suspicion of a genetic abnormality such as a family history of aneurysm, cleft palate, retinal detachment, autism spectrum disorder or other genetic disorders, for example. Based on the physician's assessment of patient history, 84 patients were selected for genetic testing which was conducted using the Invitae Connective Tissue Disorder Gene Panel (test code # 434340) which includes a panel of 92 connective tissue genes that were analyzed by Invitae. Findings were reported as benign, a variant of uncertain significance (VUS) or pathogenic.

### 2.6 Statistical analyses

Fisher's exact, Student's *t* test, and Pearson correlation were performed using Python. All graphs and analysis were completed using Python.

## 3 Results

### 3.1 Demographics

A total of 784 patients were included in the study that had been diagnosed at the EDS Clinic with hEDS or HSD from November 1, 2019, to June 20, 2022. Of those, 568 patients (aged 16–65) had at least one ECHO in their medical record at any time (including prior to the study window) that was reviewed for the study. Demographics for patients with hEDS (Table 1) or HSD (Table 2) were very similar for individuals that had at least 1 ECHO compared to their overall disease cohort indicating that the ECHO subset was representative of the larger group of hEDS or HSD patients. Most patients with hEDS (90.0%) or HSD (95.7%) were female (Tables 1, 2). Additionally, the majority of patients with at least 1 ECHO were 25–34.9 years old, White (>90%) and not Hispanic or Latinx (90%) (Tables 1, 2). The majority of patients were diagnosed with HSD (67.9%, *n* = 398) vs. hEDS (29.1%, *n* = 170). Patients with hEDS had a higher number of average Features A (∼5) than HSD patients (∼2), which is part of the diagnostic criteria (2), but similar average Beighton scores (hEDS ∼6 vs. HSD ∼5) (Tables 1, 2). For those with Feature A characteristics, most were similar between patients with hEDS and HSD except for mild skin hyperextensibility which was more prominent in patients with hEDS (Tables 1, 2).

**Table 1:** Demographics and clinical features of female and male hEDS patients diagnosed at the EDS clinic (*n* = 233) and the subsample that had at least 1 echocardiogram data available (*n* = 170).

Sample	hEDS^a^ only (*n* = 233) % (*n*)	With at least 1 ECHO (*n *= 170) % (*n*)
Patient sex		
Female	89.7% (209)	90.0% (153)
Male	10.3% (24)	10.0% (17)
Current age		
16–24.9	27.9% (65)	26.5% (45)
25–34.9	25.3% (59)	30.0% (51)
35–44.9	24.5% (57)	21.8% (37)
45–54.9	13.7% (32)	14.1% (24)
55–64.9	6.9% (16)	5.3% (9)
≥65	0.4% (1)	0.6% (1)
Race		
American Indian/Alaska Native	3.0% (7)	4.1% (7)
Asian	0.4% (1)	0.6% (1)
Black or African American	4.7% (11)	5.9% (10)
Native Hawaii/Pacific Islander	0.4% (1)	0.6% (1)
White	94.4% (220)	92.9% (158)
Other	2.2% (5)	2.4% (4)
Choose not to disclose/Unknown	2.2% (5)	2.4% (4)
Ethnicity		
Hispanic or Latino	5.6% (13)	7.7% (13)
Not Hispanic or Latino	92.3% (215)	90.0% (153)
Choose not to disclose/Unknown	2.2% (5)	2.4% (4)
Average Total Features A	4.94; SD = 1.18 (217)	4.96; SD = 1.23 (158)
Female	4.99; SD = 1.16 (195)	5; SD = 1.22 (142)
Male	4.5; SD = 1.31 (22)	4.63; SD = 1.32 (16)
Top 3 features A		
Unusually soft or velvety skin	85.0% (198)	84.7% (144)
Mild skin hyperextensibility	74.7% (174)	75.9% (129)
Bilateral piezogenic papules of the heel	72.5% (169)	72.4% (123)
Average Beighton Score	6.18; SD = 1.51 (225)	6.14; SD = 1.46 (164)
Female	6.21; SD = 1.50 (202)	6.19; SD = 1.46 (148)
Male	5.87; SD = 1.57 (23)	5.69; SD = 1.40 (16)

^a^ hEDS, hypermobile Ehlers-Danlos syndrome; HSD, hypermobility spectrum disorders; SD, standard deviation.

**Table 2:** Demographics and clinical features of female and male HSD patients diagnosed at the EDS clinic (*n* = 551) and the subsample that had at least 1 echocardiogram data available (*n* = 398).

Sample	HSD^a^ only (*n *= 551) % (*n*)	With at least 1 ECHO (*n *= 398) % (*n*)
Patient sex		
Female	96.4% (531)	95.7% (381)
Male	3.6% (20)	4.3% (17)
Current age		
16–24.9	28.7% (158)	27.6% (110)
25–34.9	26.7% (147)	27.1% (108)
35–44.9	23.2% (128)	23.4% (93)
45–54.9	14.2% (78)	14.8% (59)
55–64.9	5.6% (31)	5.8% (23)
≥65	0.4% (2)	0.3% (1)
Race		
American Indian/Alaska Native	1.3% (7)	1.3% (5)
Asian	1.3% (7)	1.0% (4)
Black or African American	1.8% (10)	1.3% (5)
Native Hawaii/Pacific Islander	0.0% (0)	0.0% (0)
White	95.5% (526)	96.5% (84)
Other	2.5% (14)	2.3% (9)
Choose not to disclose/Unknown	0.5% (3)	0.8% (3)
Ethnicity		
Hispanic or Latino	8.5% (47)	7.5% (30)
Not Hispanic or Latino	89.3% (492)	90.0% (358)
Choose not to disclose/Unknown	2.2% (12)	2.5% (10)
Average Total Features A	2.06; SD = 1.05 (530)	2.08; SD = 1.06 (382)
Female	2.06; SD = 1.05 (510)	2.08; SD = 1.05 (365)
Male	2.05; SD = 1.12 (*n* = 20)	1.94; SD = 1.16 (17)
Top 3 features A		
Bilateral piezogenic papules of the heel	46.1% (254)	46.0% (183)
Unexplained striae distensae or rubae at the back, groins, thighs, breasts and/or abdomen in adolescents, men or pre-pubertal women without a history of significant gain or loss of body fat or weight	39.4% (217)	41.5% (165)
Unusually soft or velvety skin	34.3% (189)	33.4% (133)
Average Beighton Score	5.21; SD = 1.21 (541)	5.21; SD = 1.17 (390)
Female	5.21; SD = 1.21 (521)	5.21; SD = 1.18 (373)
Male	5.2; SD = 1.00 (20)	5.24; SD = 1.00 (17)

^a^ hEDS, hypermobile Ehlers-Danlos syndrome; HSD, hypermobility spectrum disorders; SD, standard deviation.

### 3.2 Aortic root dilatation

**Figure 1:**
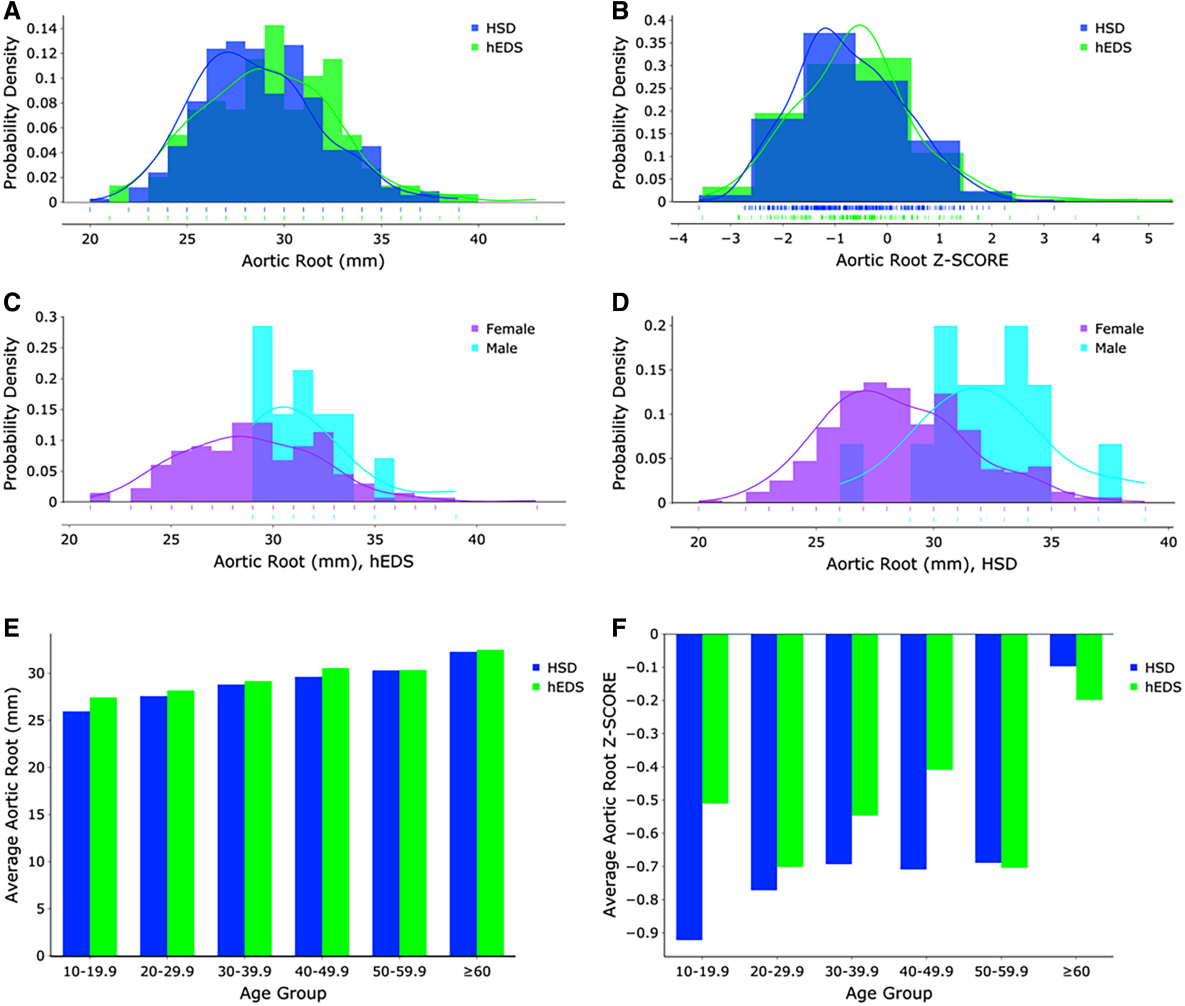
(**A**) Aortic root measurements (mm) and (**B**) z-score distributions for hEDS (blue) and HSD (green) patients (*n* = 481). Aortic root measurements (mm) for (**C**) hEDS and (**D**) HSD females (purple) and males (turquoise). (**E**) Aortic root measurements (mm) and (**F**) z-score distributions for hEDS (blue) and HSD (green) patients according to age group.

Of the 568 hEDS and HSD patients with at least one ECHO, 481 (84.7%) had an aortic root diameter measurement (Tables 3, 4, Supplementary Figure S1). The average aortic root z-score after Devereux z-score normalization at the first ECHO for patients with hEDS was −0.58 (*n *= 148), and for HSD was −0.73 (*n* = 333), indicating that most patients did not have dilated aortic roots. There was no significant difference in average aortic root diameter between patients with HSD (28.5 mm, *n *= 333) compared to hEDS (29.1 mm, *n* = 148, *p* = 0.070) (Figure 1A) or for average aortic root z-score when HSD (−0.73, *n* = 333) was compared to hEDS (−0.58, *n* = 148, *p* = 0.185) (Figure 1B). In a study by Asher et al. the average root diameter after Devereux z-score normalization in EDS patients was −0.97 ± 1.2 at the time of the first ECHO indicating that our findings are similar to previous reports (5). We had 101 patients from the EDS Clinic who were not diagnosed with hEDS or HSD that had an available ECHO and 77 with recorded aortic root measurements. Note that these were not healthy individuals. These patients attended the EDS Clinic because of suspicion for hEDS or HSD but were not diagnosed with either condition. They had many comorbidities based on the intake questionnaire. We found that there was no statistically significant difference in the average aortic root diameter between patients with hEDS (*p* = 0.076, *t* test) or HSD (*p* = 0.425, *t* test) and this group of patients.

**Table 3:** Aortic root measurement and Z-SCORE by age group for patients with hEDS (*n* = 148).

Age	*n*	Mean	Standard deviation	Minimum	Maximum
Aortic root measurement (mm)					
10–19.9	14	27.4	3.2	21	32
20–29.9	51	28.2	3.3	21	38
30–39.9	38	29.2	3.8	23	39
40–49.9	27	30.6	3.6	26	43
50–59.9	14	30.4	2.6	25	34
≥60	4	32.5	1.3	31	34
Aortic root Z-SCORE					
10–19.9	14	−0.51	1.03	−2.83	1.38
20–29.9	51	−0.70	1.16	−3.54	3.60
30–39.9	38	−0.55	1.37	−2.85	2.89
40–49.9	27	−0.41	1.43	−2.84	4.80
50–59.9	14	−0.70	0.86	−2.30	0.58
≥60	4	−0.20	0.42	−0.71	0.28

**Table 4:** Aortic root measurement and Z-SCORE by age group for patients with HSD (*n* = 333).

Age	*n*	Mean	Standard deviation	Minimum	Maximum
Aortic root measurement (mm)					
10–19.9	29	26.0	2.8	20	33
20–29.9	114	27.6	2.7	22	37
30–39.9	92	28.8	3.0	23	39
40–49.9	59	29.6	3.3	24	37
50–59.9	32	30.3	3.2	25	39
≥60	7	32.3	2.8	28	35
Aortic root Z-SCORE					
10–19.9	29	−0.92	0.88	−2.38	1.04
20–29.9	114	−0.77	0.97	−2.64	2.25
30–39.9	92	−0.69	1.10	−3.61	3.20
40–49.9	59	−0.71	1.14	−2.71	1.47
50–59.9	32	−0.69	1.13	−2.52	1.19
≥60	7	−0.10	1.05	−1.72	0.94

As expected, male patients with hEDS had significantly larger average aortic root diameters compared to females (females *n* = 133, diameter 28.85 mm, z-score −0.58; males *n* = 15, diameter 31.53 mm, z-score −0.52, *p* = 0.005) (Figure 1C). Similarly male patients with HSD also had significantly larger average aortic root diameters compared to females (females *n *= 317, diameter 28.32 mm, z-score −0.73; males *n* = 16, diameter 32.13 mm, z-score −0.68, *p* < 0.0001) (Figure 1D). However, there was no significant difference by sex in average z-scores in patients with hEDS (*p* = 0.851) or HSD (*p* = 0.854) which is expected because the z-score controls for larger body size (typically larger in males) and increased size with age (12). Also as expected, aortic root diameter increased with increasing age in patients diagnosed with hEDS or HSD (Tables 3, 4, Figures 1E,F, Supplementary Figure S2) (13). The largest average aortic root diameter and average z-score were found in patients with hEDS or HSD who were 60 years or older (Tables 3, 4, Figures 1E,F, Supplementary Figure S2), similar to results of a previous study of aortic root diameter in patients with hEDS by age (5).

We did not find a significant relationship between aortic root diameter and self-reported cardiac comorbidities from our intake questionnaire in patients diagnosed with hEDS except for a borderline significant relationship for bradycardia (*p* = 0.085) (Table 5). However, bradycardia was significant when examining the average aortic root z-score (*p* = 0.034) in patients with hEDS along with brain aneurysm (*p* = 0.015) (Table 6). We found that patients diagnosed with HSD that self-reported chest pain (*p* = 0.044) or hypertension (*p* = 0.008) had significantly larger aortic root diameters while white coat hypertension was borderline significant (*p* = 0.072) (Table 7). HSD patients with dysautonomia (*p* = 0.019) had significantly larger average aortic root z-scores (i.e., less negative) and patients who self-reported chest pain were borderline significant for larger z-scores (*p* = 0.070) (Table 8). We also found an absence of valvular complications in patients with hEDS or HSD with aortic root dilation. Almost all aortic root dilation patients had trivial regurgitation in their mitral, tricuspid, or pulmonary valve.

**Table 5:** Aortic root measurement averages by frequency of self-reported cardiac comorbidities for patients with hEDS (*n* = 148).

Cardiac History	Yes (*n*)	Yes: mean aortic root measurement (mm)	No (*n*)	No: mean aortic root measurement (mm)	*p* value^a^
Hypotension/low blood pressure	97	29.5	51	28.9	0.311
Tachycardia	52	28.8	96	29.3	0.460
Chest pain	49	29.0	99	29.2	0.803
Dysautonomia	41	29.0	107	29.2	0.779
Orthostatic hypotension	34	29.1	114	29.1	0.900
Arrhythmia	19	28.3	129	29.2	0.214
Heart valves issues	16	28.7	132	29.2	0.635
Hypertension	14	29.4	134	29.1	0.731
White coat hypertension	12	29.0	136	29.1	0.872
POTS- postural orthostatic tachycardia syndrome	13	28.4	135	29.2	0.468
**Bradycardia**	**10**	**30** **.** **7**	**138**	**29** **.** **0**	**0** **.** **085**
Pectus excavatum	8	31.1	140	29.0	0.106
Atrial fibrillation	4	26.8	144	29.2	0.407
Aneurysm/dissection of aorta in chest cavity	2	31.0	146	29.1	0.514
Brain aneurysm	2	25.5	146	29.2	0.236
Pectus carinatum	1	34.0	147	29.1	–
Myocarditis/pericarditis	1	32.0	147	29.1	–
Aneurysm/dissection of abdominal aorta (AA)	1	29.0	147	29.1	–
Diabetes- type 2	0	–	148	29.1	–
Other	23	28.6	125	29.2	0.509
No issues	13	28.8	135	29.2	0.754
Unknown	19	29.7	129	29.0	0.445

^a^ *P* values result from independent samples *t*-test comparing aortic root measurement averages between patients who answered “yes” and patients who answered “no” for a given cardiac condition.

Bold indicates borderline significant finding.

**Table 6:** Aortic root Z-SCORE averages ordered by frequency of self-reported cardiac comorbidities in hEDS patients with aortic root measurements (*n* = 148).

Cardiac History	Yes (*n*)	Yes: mean Z-SCORE	No (*n*)	No: mean Z-SCORE	*p* value^a^
Hypotension/low blood pressure	97	−0.46	51	−0.64	0.348
Tachycardia	52	−0.58	96	−0.57	0.971
Chest pain	49	−0.53	99	−0.60	0.750
Dysautonomia	41	−0.46	107	−0.62	0.460
Orthostatic hypotension	34	−0.57	114	−0.58	0.979
Arrhythmia	19	−0.79	129	−0.54	0.321
Heart valves issues	16	−0.91	132	−0.54	0.286
Hypertension	14	−0.77	134	−0.56	0.503
POTS- postural orthostatic tachycardia syndrome	13	−0.45	135	−0.59	0.747
White coat hypertension	12	−0.79	136	−0.56	0.477
**Bradycardia**	**10**	**0** **.** **14**	**138**	**−0** **.** **63**	**0** **.** **034**
Pectus excavatum	8	0.16	140	−0.62	0.178
Atrial fibrillation	4	−1.34	144	−0.56	0.315
Aneurysm/dissection of aorta in chest cavity	2	0.23	146	−0.59	0.233
**Brain aneurysm**	**2**	**−1** **.** **86**	**146**	**−0** **.** **56**	**0** **.** **015**
Aneurysm/dissection of abdominal aorta (AA)	1	−0.12	147	−0.58	–
Myocarditis/pericarditis	1	−0.27	147	−0.58	–
Pectus carinatum	1	0.28	147	−0.58	
Diabetes- type 2	0		148	−0.58	–
Other	23	−0.89	125	−0.52	0.320
No issues	13	−0.41	135	−0.59	0.679
Unknown	19	−0.52	129	−0.58	0.835

^a^ *P* values result from independent sample *t* test comparing Z-SCORE averages between patients who answered “yes” and patients who answered “no” for a given cardiac condition.

Bold indicates significant findings.

**Table 7:** Aortic root measurement averages by frequency of self-reported cardiac comorbidities for patients with HSD (*n* = 333).

Cardiac history	Yes (*n*)	Yes: mean aortic root measurement (mm)	No (*n*)	No: mean aortic root measurement (mm)	*p* value^a^
Tachycardia	107	28.4	226	28.5	0.708
Hypotension/low blood pressure	96	28.6	237	28.5	0.682
**Chest pain**	**80**	**27** **.** **9**	**253**	**28** **.** **7**	**0** **.** **044**
Dysautonomia	75	28.8	258	28.4	0.398
Orthostatic hypotension	68	28.6	265	28.5	0.839
**Hypertension**	**46**	**29** **.** **8**	**287**	**28** **.** **3**	**0** **.** **008**
Heart valves issues	31	28.9	302	28.5	0.456
Arrhythmia	43	28.6	290	28.5	0.830
**White coat hypertension**	**34**	**29** **.** **6**	**299**	**28** **.** **4**	**0** **.** **072**
Bradycardia	25	29.3	308	28.4	0.271
POTS- postural orthostatic tachycardia syndrome	17	28.5	316	28.5	0.978
Pectus excavatum	8	29.6	325	28.5	0.393
Atrial fibrillation	4	31.3	329	28.5	0.270
Brain aneurysm	4	27.8	329	28.5	0.441
Myocarditis/pericarditis	4	30.5	329	28.5	0.355
Pectus carinatum	4	29.0	329	28.5	0.676
Aneurysm/dissection of abdominal aorta (AA)	1	28.0	332	28.5	–
Aneurysm/dissection of aorta in chest cavity	0	–	333	28.5	–
Diabetes- type 2	0	–	333	28.5	–
Other	30	28.5	303	28.5	0.958
No issues	51	28.2	282	28.6	0.424
Unknown	38	28.4	295	28.5	0.912

^a^ *P* values result from independent samples *t*-test comparing aortic root measurement averages between patients who answered “yes” and patients who answered “no” for a given cardiac condition.

Bold indicates significant or borderline significant finding.

**Table 8:** Aortic root Z-SCORE averages ordered by frequency of self-reported cardiac comorbidities in HSD patients with aortic root measurements (*n* = 333).

Cardiac History	Yes (*n*)	Yes: mean Z-SCORE	No (*n*)	No: mean Z-SCORE	*p* value^a^
Tachycardia	107	−0.72	226	−0.74	0.882
Hypotension/low blood pressure	96	−0.58	237	−0.79	0.098
**Chest pain**	**80**	−**0.90**	**253**	−**0.68**	**0** **.** **070**
**Dysautonomia**	**75**	−**0.49**	**258**	−**0.80**	**0** **.** **019**
Orthostatic hypotension	68	−0.69	265	−0.74	0.696
Hypertension	46	−0.49	287	−0.77	0.124
Arrhythmia	43	−0.71	290	−0.73	0.901
White coat hypertension	34	−0.57	299	−0.75	0.433
Heart valves issues	31	−0.57	302	−0.75	0.387
Bradycardia	25	−0.65	308	−0.74	0.671
POTS- postural orthostatic tachycardia syndrome	17	−0.39	316	−0.75	0.260
Pectus excavatum	8	−0.45	325	−0.74	0.581
Atrial fibrillation	4	−0.06	329	−0.74	0.462
Brain aneurysm	4	−1.08	329	−0.73	0.486
Myocarditis/pericarditis	4	−0.10	329	−0.74	0.384
Pectus carinatum	4	−0.88	329	−0.73	0.779
Aneurysm/dissection of abdominal aorta (AA)	1	−1.78	332	−0.73	–
Aneurysm/dissection of aorta in chest cavity	0	–	333	−0.73	–
Diabetes- type 2	0	–	333	−0.73	–
Other	30	−0.77	303	−0.73	0.790
No issues	51	−0.79	282	−0.72	0.637
Unknown	38	−0.78	295	−0.72	0.774

^a^ *P* values result from independent sample *t* test comparing Z-SCORE averages between patients who answered “yes” and patients who answered “no” for a given cardiac condition.

Bold indicates significant or borderline significant finding.

We found that 6 out of 481 patients in this study had aortic root dilation based on a z-score >2.0 (Table 9). All six were female and were under 45 years of age at first ECHO, with four of the six patients diagnosed with hEDS and two with HSD (Table 9). Based on these findings, the prevalence of aortic root dilation was 2.7% in patients with hEDS (*n* = 148) and 0.6% in patients with HSD (*n* = 333) (Table 10). Patients with a dilated aortic root who underwent a second ECHO (*n* = 2) did not display an increase in either aortic root z-score or aortic root diameter measurement (Table 9). Self-reported cardiac comorbidities are listed for the 6 patients with aortic root dilation in Table 11. Two of the six patients with aortic root dilation self-reported having postural orthostatic tachycardia syndrome (POTS) (one with hEDS and one with HSD) (Table 11). This compared to 13 hEDS patients (*n* = 148, 8.8%), 17 HSD patients (*n* = 333, 5.1%) and 2 patients that were not diagnosed with hEDS or HSD (*n* = 101, 2%) in this study (Supplementary Table S1). POTS did not occur more often in hEDS (*p* = 0.311, Fisher's Exact test) or HSD (*p* = 0.100, Fisher's Exact test) patients with aortic root dilation. We had only three patients with a z-score greater than 3, indicating clinically important dilation (Table 9, Supplementary Table S2). When we examined whether these patients had a self-reported family history of heritable aortic disease, we found that one patient reported a family history of brain aneurysm and another patient reported a family history of abdominal aortic aneurysm (Supplementary Table S2). Of these patients only the patient with HSD and a z-score >3.0 had the connective tissue gene panel run, but the results were negative (Table 9).

**Table 9:** Aortic root Z-SCORE and measurements at first and second echocardiogram for all patients with a dilated aortic root^a^ (*n* = 6).

#	Sex	hEDS/HSD^b^	BSA	1st ECHO Age	1st ECHO: aortic root (mm)	1st ECHO: Z-SCORE	2nd ECHO: Age	2nd ECHO: aortic root (mm)	2nd ECHO: Z-SCORE
1	F	hEDS	1.69	42	43	4.8	43	40	3.6
2	F	hEDS	1.54	29	38	3.6	–	–	–
3	F	HSD^c^	1.81	38	39	3.2	–	–	–
4	F	hEDS	1.63	34	37	2.9	35	34	1.7
5	F	hEDS	1.7	35	36	2.3	–	–	–
6	F	HSD	2.13	27	37	2.2	–	–	–

^a^ Aortic root dilation defined as a Z-score >2.0.

^b^ BSA, body surface area; F, female; hEDS, hypermobile Ehlers-Danlos syndrome; HSD, hypermobility spectrum disorders.

^c^ Patient received connective tissue genetic testing and results were negative.

**Table 10:** Aortic root dilation prevalence in patients with hEDS or HSD (*n* = 481).

	hEDS (*n*)	% hEDS	HSD (*n*)	% HSD
Dilated^a^	4	2.7%	2	0.6%
Not Dilated	144	97.3%	331	99.4%
Total	148	100.0%	333	100.0%

^a^ Aortic root dilation defined as a Z-Score >2.0.

**Table 11:** Self-reported cardiac history for patients with hEDS (*n* = 4) or HSD (*n* = 2) with aortic root dilation (ARD).

Cardiac History^a^	Frequency of cardiac comorbidities in hEDS patients with ARD^b^	Frequency of cardiac comorbidities in HSD patients with ARD
Chest pain	1	
Dysautonomia	1	1
Hypertension		1
Hypotension/low blood pressure		1
Pectus excavatum	1	
POTS (postural orthostatic tachycardia syndrome)	1	1
Tachycardia		1
White coat hypertension		1
Other	1	
No issues	1	

^a^ Self-reported cardiac history obtained from Intake Questionnaire.

^b^ ARD, aortic root dilation; BP, blood pressure; hEDS, hypermobile Ehlers-Danlos syndrome; HSD, hypermobility spectrum disorders.

### 3.3 Valvular regurgitation

The majority of hEDS patients were found to have trivial mitral (71.3%), tricuspid (81.2%) or pulmonary (57.4%) regurgitation at their first ECHO (Table 12). A similar result was found for patients diagnosed with HSD who had trivial mitral (72.7%), tricuspid (84%) or pulmonary (63.9%) regurgitation at their first ECHO (Table 12). Less than 10% of hEDS patients were found to have mild aortic (2.4%), mitral (4.8%), tricuspid (8.8%) or pulmonary (10.5%) regurgitation at first ECHO (Table 12). Similarly, less than 10% of HSD patients were found to have mild aortic (0.5%), mitral (5.4%), tricuspid (8.1%) or pulmonary (10.3%) regurgitation at first ECHO (Table 12). Mild aortic regurgitation occurred more often in patients diagnosed with hEDS (2.4%) compared to HSD (0.5%). Around 2% or less of patients with hEDS or HSD had thickened or sclerotic valves by ECHO (Supplementary Table S3).

**Table 12:** Valve regurgitation by severity in patients with hEDS (*n* = 170) or HSD (*n* = 398).

Type^a^	None^b^	Trivial	Mild	Mild-Moderate	Moderate
hEDS					
Aortic Regurgitation	152 (91.6%)	10 (6.02%)	4 (2.4%)	0	0
Mitral Regurgitation	38 (22.8%)	119 (71.3%)	8 (4.8%)	1 (0.6%)	1 (0.6%)
Tricuspid Regurgitation	16 (9.4%)	138 (81.2%)	15 (8.8%)	1 (0.6%)	0
Pulmonary Regurgitation	52 (32.1%)	93 (57.4%)	17 (10.5%)	0	0
HSD					
Aortic Regurgitation	354 (91.5%)	31 (8%)	2 (0.52%)	0	0
Mitral Regurgitation	86 (21.9%)	285 (72.7%)	21 (5.4%)	0	0
Tricuspid Regurgitation	30 (7.6%)	331 (84%)	32 (8.1%)	1 (0.25%)	0
Pulmonary Regurgitation	96 (25.5%)	241 (63.9%)	39 (10.3%)	1 (0.27%)	0

^a^ All rows do not add up to 170 due to missing data for some patients.

^b^ Data lists number of patients (percent).

### 3.4 Mitral valve prolapse

The prevalence of diagnosed mitral valve prolapse (MVP) in patients with hEDS was 6/170 (3.5%) vs. 7/398 (1.8%) in patients with HSD. There was no significant difference in prevalence for MVP between patients diagnosed with hEDS vs. HSD (*p* = 0.224). All 13 cases of MVP that were identified (plus 2 possible cases making 15) occurred in women with hEDS or HSD who ranged in age from 18 to 70 (Table 13, Supplementary Figure S3). Bi-leaflet MVP was the most common type of MVP observed in hEDS or HSD women (Supplementary Table S4). Cardiac conditions associated with MVP are listed in Supplementary Table S5. The severity of MVP was noted as trivial in 8 patients, mild in 5 patients and moderate in 2 patients (Table 13, Supplementary Table S6). There were two female patients with HSD who had trivial MVP that also developed arrhythmia, and two patients with mild regurgitation that had thickened mitral valves (Supplementary Tables S5–S7). None of the patients with hEDS or HSD with MVP had severe regurgitation.

**Table 13:** Severity of mitral valve regurgitation (MVR) and impressions for all possible mitral valve prolapse (MVP) patients (*n* = 15).

Patient	hEDS or HSD	Age at ECHO	MVR^a^	Impression	LV diastolic dysfunction
1	hEDS	42	Trivial	Increased mitral valve deceleration time and decreased E wave velocity for age group	Normal
2	HSD	41	Trivial	Normal ranges	Normal
3	hEDS	38	Trivial	Increased mitral valve deceleration time and reduced E/A wave ratio for age group	Grade 1/4, consistent with low to normal left ventricular filling pressure
4	hEDS	49	Trivial	Normal ranges	Normal
5	HSD	24	Trivial	Increased A wave velocity	Indeterminate
6	hEDS	70	Moderate	Increased E and A wave velocities	Indeterminate
7	HSD	57	Mild	Increased A wave velocity	Normal
8	hEDS	18	Mild	Normal Ranges	Normal
9	hEDS	57	Mild-Moderate	Normal Ranges	NR
10	hEDS	23	Mild	NR	Indeterminate
11	HSD	31	Trivial	Normal Ranges	Normal
12	HSD	34	Trivial	Normal Ranges	NR
13	HSD	52	Mild	Increased A wave velocity	Normal
14	HSD	46	Mild	Normal Ranges	Normal
15	HSD	31	Trivial	Normal Ranges	Normal

^a^ hEDS, hypermobile Ehlers-Danlos syndrome; HSD, hypermobility spectrum disorders; ECHO, echocardiogram; MVR, mitral valve regurgitation; LV, left ventricle; NR, not reported.

### 3.5 Genetic testing

Genetic testing was conducted on 84 patients from the EDS Clinic (36 with hEDS and 48 with HSD) during the study window. No connective tissue-related genes have been identified as causative/pathogenic in patients with hEDS or HSD and so genetic testing is not indicated or routinely conducted in these patients. Selection for obtaining genetic testing is based on family and medical history. A finding of a genetic variant was identified in 16 (19%) patients, but all were variants of uncertain significance (VUS) (Supplementary Table S8). Twelve of the 16 patients with genetic variants were diagnosed with HSD (75%) vs. hEDS (25%). Six of the 16 patients (37.5%) with a genetic variant had a cardiac abnormality including issues like MVP and mild aortic regurgitation (Supplementary Table S8). Four patients had genetic variants in collagen genes, but only one of these patients had mild pulmonary valve regurgitation (Supplementary Table S8). Three patients had polymorphisms in filamin A (*FLNA*) with one patient having mild aortic regurgitation and another patient having mitral valve anterior leaflet prolapse, moderate mitral valve regurgitation and mild aortic and pulmonary regurgitation. And two patients were carriers for variants in lysl hydrolase (*PLOD1*), which is associated with the kyphoscoliotic form of EDS (Supplementary Table S8). Some variants listed in Supplementary Table S8 are not specifically related to connective tissue issues, aortic or valve disease such as *HFE* and *MYH7*.

## 4 Discussion

Hypermobile forms of EDS, which are the most common subtype of EDS, are inherited in an autosomal dominant manner. The genetic variant(s) contributing to hEDS and HSD are unknown. This study aimed to understand the prevalence of cardiovascular complications in adult patients with hEDS vs. HSD using a large well-defined cohort from the Mayo EDS Clinic diagnosed using the 2017 classification. Previous studies had combined data for hEDS and HSD or used a combination of diagnoses including classical EDS. A number of previous studies found that 6%–21% of patients with symptomatic hypermobility are at risk of ARD or MVP (6, 7), which prompted the requirement to examine ARD and MVP in the 2017 diagnostic process. However, other studies have reported a low prevalence similar to our results (5, 8, 14).

We found in this study that the overall prevalence of ARD in patients with hEDS was 2.7% and with HSD was 0.6% (Table 10). The prevalence of MVP in patients in this study with hEDS was 3.5% or HSD was 1.8%, similar to the prevalence of MVP in the general population of 2.7% (15). In contrast, Rashed et al. reported a prevalence of ARD of 15.2% and MVP of 7.5% in patients diagnosed with hEDS and HSD combined (7). Rashed et al. found a significant difference between patients, with ARD and MVP being higher in patients diagnosed with hEDS as expected based on the diagnostic criteria. However, we found no significant difference between hEDS and HSD patients in our study. Although the 2017 diagnostic criteria require an assessment of ARD and MVP, confirming a diagnosis of hEDS does not require the presence of these cardiovascular complications. Thus, our findings from a large cohort of well-defined hEDS and HSD patients indicate that the prevalence of MVP is similar to the general population and similar to the findings of several other studies (5, 8, 14), but those studies did not evaluate patients only using the 2017 diagnostic criteria. In contrast, Rashed et al. used the 2017 diagnostic criteria, mainly in adults (94%) with similar methods to evaluate ARD as our study (7). Reasons that we may have found a different prevalence than Rashed et al. is that we examined a higher number of patients, and patients were referred in the Rashed study to a specialty Cardiovascular Genetics Program which may have biased toward patients with more cardiovascular issues (7).

We found very few patients with hEDS or HSD that had a dilated aortic root in this study. Additionally, we did not find a significant difference in z-score between patients with hEDS or HSD, which is perhaps not surprising considering the high degree of overlap in many clinical features we and others have found between the two diagnoses (5, 10, 16). We were not using a pediatric z-score but one that incorporated body size area (BSA) as recently recommended for adults (12, 13). There was however no significant difference in aortic root measurements in our study between patients who were diagnosed with hEDS or HSD. Finding a difference in the z-score for an individual patient reveals the limitations of z-score normalization using the Boston and Devereux formulas, as highlighted by Ritter et al. (6). Because of this, clinical interpretation of a patient's z-score should be carefully considered. The average aortic root diameter for males with hEDS or HSD in this study was greater than the average reported by Rashed et al. (7). However, Rashed et al. had a lower number of male patients (*n *= 25) than our study (*n* = 34) and also reported 4 out of 25 male patients with aortic root dilatation whereas no males had dilatation in our study (7). This disparity can be explained by small sample sizes for males. For females, the average aortic root measurement for those with dilatation in our study was also greater. However, increasing prevalence of aortic dilatation by age was observed in both studies.

Although MVR and MVP are known to increase with age (17), we found that 11 out of 15 (73%) confirmed or suspected cases of MVP occurred in patients with hEDS or HSD that were under 50 years of age (Table 13). Thus, our study does not suggest that MVP occurs more often in older patients with hEDS or HSD. However, this may be related to the fact that hEDS and HSD are typically diagnosed in patients under age 50 and that patients experience joint stiffening with age (reduced hypermobility), so that a patient that was diagnosed when young may not fulfill the diagnostic criteria when they are past age 50.

Genetic testing was conducted on a subset of patients in this study based on family and/or medical history. Because no genetic variant has been identified for hEDS or HSD, conducting genetic testing on all patients is not currently indicated. Only a small percentage of those tested were found to have genetic variants, all of which were VUS. Rashed et al. also examined whether there was a relationship between genetic variants and cardiac issues in patients with hEDS/HSD (7). They found that of 22 patients with ARD and five patients with extra-aortic manifestations who underwent genetic testing, results were either negative or identified as a VUS like our findings (7). In our study, there was a low number of genetic variants identified for patients with hEDS or HSD, consistent with the literature. Research is needed to investigate connective tissue VUS identified in patients with hEDS and HSD to determine whether they are related to disease severity in general or to cardiovascular outcomes. Although genetic testing is not currently indicated for patients with hEDS or HSD, certain indicators in the family or medical history may warrant genetic testing and echocardiology evaluation.

The strengths of this study are a large sample size of well characterized hEDS and HSD patients, availability of a data collection service to collect ECHO data from the medical record, independent measurement of aortic root diameters, and a large amount of information available about patients including cardiac history from an intake questionnaire. Another benefit of the study was a unique setting with a single provider who is an EDS specialist who evaluated the patients at an EDS Clinic, heart disease specialists to interpret ECHO readings, and basic researchers to assess trends in the data. Limitations of the study include a self-reported assessment of cardiovascular history and the small number of males with a diagnosis of hEDS or HSD. Another limitation is the lack of genetic screening for all patients and the lack of healthy controls for comparison to hEDS/HSD. We showed previously that 64% of the individuals that attended our EDS Clinic but were not diagnosed with hEDS or HSD were diagnosed with fibromyalgia (10). A study by Kozanoglu et al. found that females with joint hypermobility and fibromyalgia were 9× more likely to have MVP than patients with fibromyalgia alone (18). Our findings suggest that MVP may not occur at a high rate in this group of patients at our EDS Clinic, but further research is needed. Our previous study showed that patients that are not diagnosed with hEDS or HSD at our EDS Clinic have significantly fewer symptoms and comorbidities compared to hEDS and HSD patients, suggesting that they may not be the same patient groups (18). However, studies comparing rates of ARD and MVP in patients with hEDS and HSD compared to normal healthy controls are needed.

This study has implications for clinicians. Our findings suggest that an ECHO may not be routinely needed in all newly diagnosed patients with hEDS or HSD because only a small number of cardiac defects were identified, and those complications were predominantly mild. Additionally, cardiac testing, which may not have been conducted at the time of diagnostic evaluation, may delay the diagnosis for patients using the 2017 diagnostic criteria, which requires assessment of ARD and/or MVP. However, determining whether a patient has ARD and/or MVP can only be assessed by ECHO. Even though there is a low prevalence of events in patients with hEDS and HSD, if ECHOs are not routinely performed these cardiovascular complications could be missed. Although our study is the largest to date to examine this issue, more research is needed with larger numbers because some recent studies such as Rashed et al. have found significant numbers of patients with hEDS/HSD with ARD and MVP (7). Future iterations of the hEDS/HSD diagnostic criteria should be revised related to cardiovascular findings based on the most recent research. Overall, our findings indicate that cardiac defects in patients with hEDS/HSD are similar to the general population.

## 5 Conclusions

Patients with hEDS and HSD in our study had few cardiovascular complications suggesting that routine echocardiography as part of the initial workup may not be warranted. However, other current studies using similar criteria have found a higher prevalence of ARD and MVP in patients with hEDS and HSD than our study and the general population indicating that more research is needed. Overall, we did not observe a difference in cardiovascular complications in patients with hEDS vs. HSD, which were mild, infrequent and similar to the general population.

## Data Availability

The original contributions presented in the study are included in the article/**Supplementary Material**, further inquiries can be directed to the corresponding author.
