# Supplementary material for: Cardiac defects of hypermobile Ehlers-Danlos syndrome and hypermobility spectrum disorders: a retrospective cohort study
Source: Front Cardiovasc Med. 2024 Mar 18;11:1332508. doi: 10.3389/fcvm.2024.1332508 (PMC10982405; doi:10.3389/fcvm.2024.1332508)
Supplement: Supplementary file 1 [file Datasheet1.docx]

Supplementary Material

Cardiac Defects of Hypermobile Ehlers-Danlos Syndrome and Hypermobility Spectrum Disorders: A Retrospective Cohort Study

**Dacre R. T. Knight, MD^1*$#^, Katelyn A. Bruno, PhD^1,2,3*$^, Ayush Singh, BS^1*^, Bala Munipalli, MD^1^, Shilpa Gajarawala,** **PA-C^1^, Mahima Solomon, MBBS^1^, S. Christian Kocsis,^2^ Ashley A. Darakjian^2^, Angita Jain, MBBS^2,4,5^, Emily R. Whelan^2,4,5^, Archana Kotha, MBBS^2^,** **David J. Gorelov^2^, Sabrina D. Phillips, MD^2$^, DeLisa Fairweather, PhD^1,2,4,6$^**

^1^Department of General Internal Medicine, Mayo Clinic, Florida, USA

^2^Department of Cardiovascular Medicine, Mayo Clinic, Florida, USA

^3^Division of Cardiovascular Medicine, Department of Medicine, University of Florida, Gainesville, Florida, USA

^4^Center for Clinical and Translational Science, Mayo Clinic, Rochester, Minnesota, USA

^5^Mayo Clinic Graduate School of Biomedical Sciences, Mayo Clinic, Rochester, Minnesota, USA

^6^Department of Immunology, Mayo Clinic, Jacksonville, Florida, USA

***First authorship:** DRTK, KAB, AS share first authorship.

**$Equal contribution and senior authorship:** DRTK, KAB, SDP, DF contributed equally to this work and share senior authorship.

**#** **Correspondence:** Dacre Knight

[Knight.Dacre@mayo.edu](mailto:Knight.Dacre@mayo.edu)

# Supplementary Figures and Tables

## Supplementary Figures


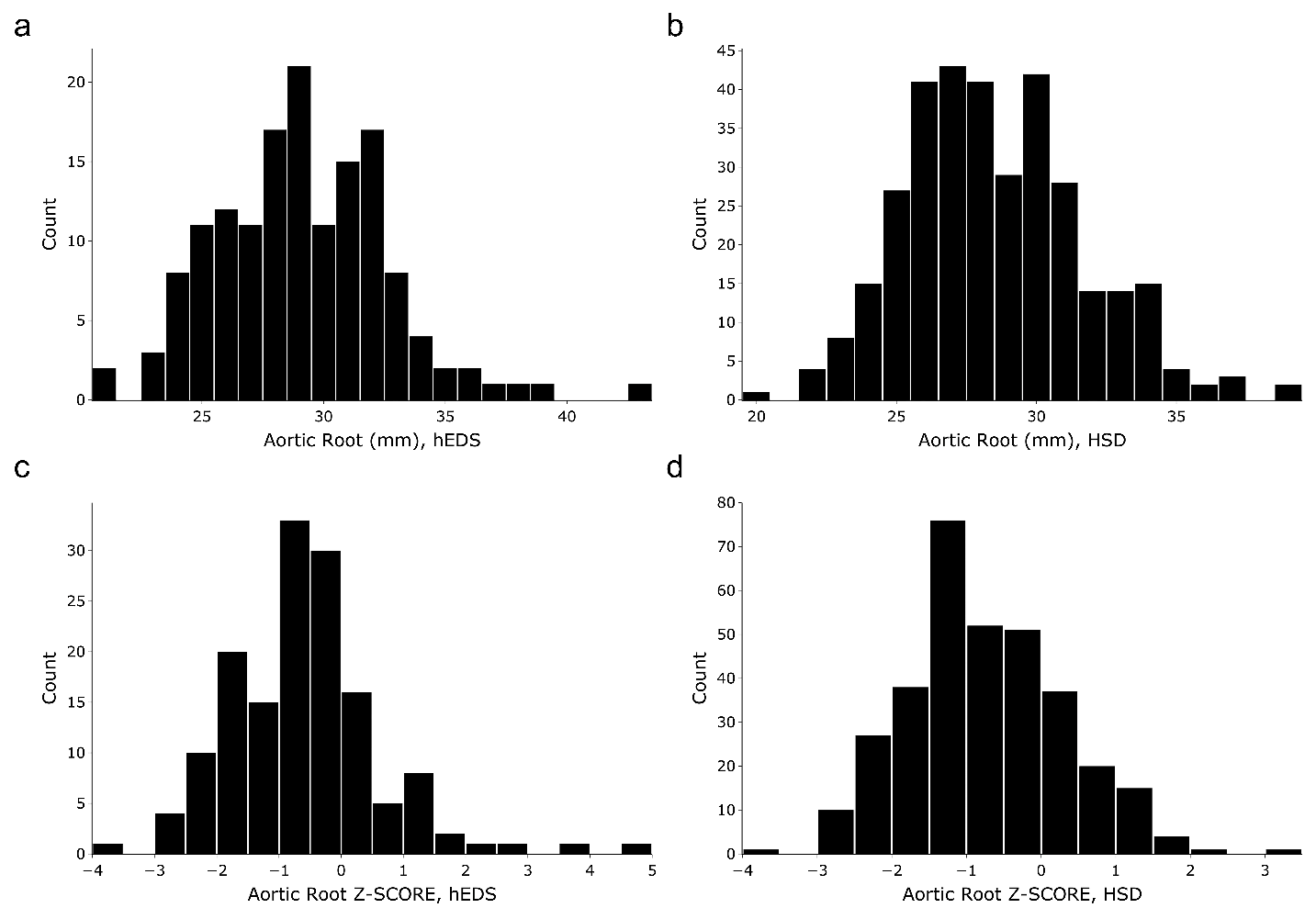


**Supplementary Figure 1.** Aortic root measurements (mm) for patients with a) hEDS or b) HSD and z-score distributions for patients with c) hEDS or d) HSD (*n* = 481).


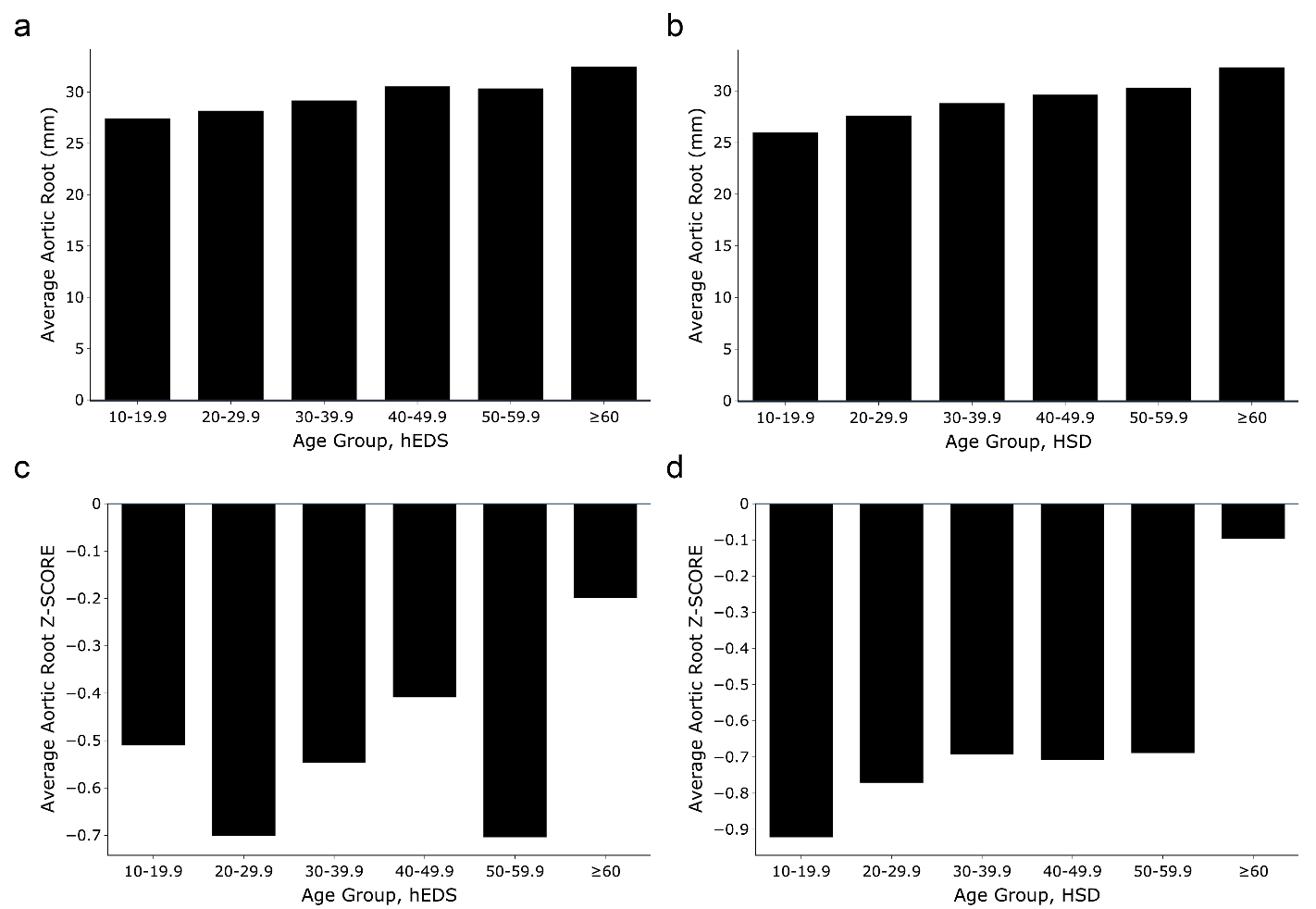


**Supplementary Figure 2.** Aortic root measurements (mm) for patients by age with a) hEDS or b) HSD and z-score distributions for patients by age with c) hEDS or d) HSD (*n* = 481).


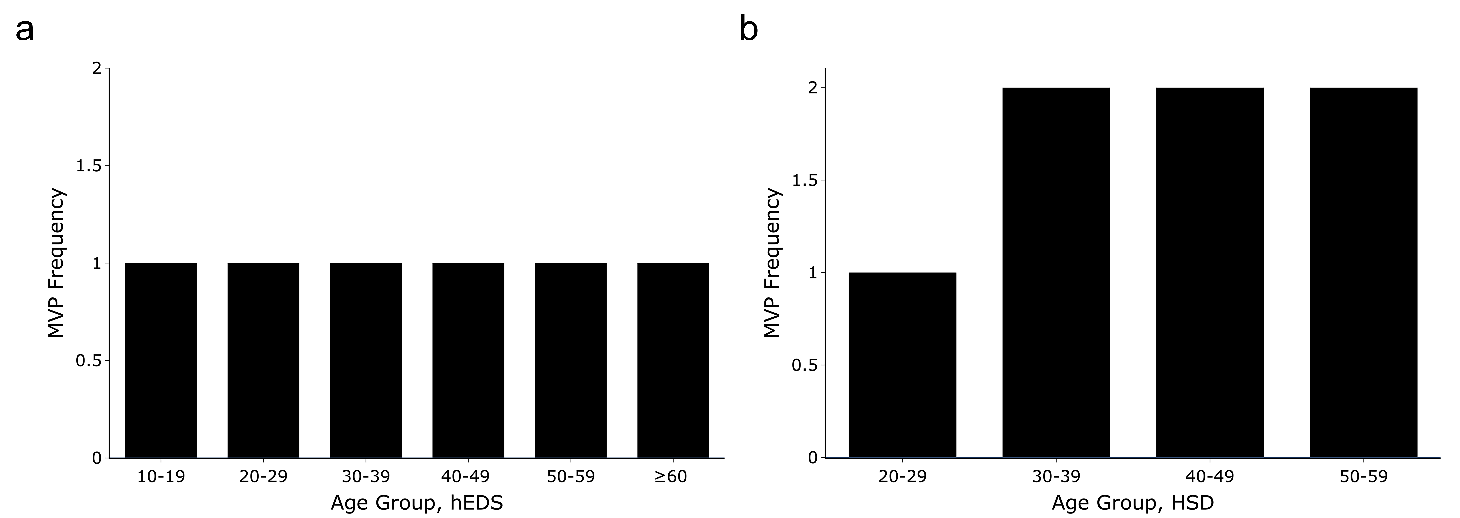


**Supplementary Figure 3.** Mitral valve prolapse (MVP) frequency by age in patients with a) hEDS or b) HSD (*n* = 13).

**1.2 Supplementary Tables**

**Supplementary Table 1.** Prevalence of POTS in the entire cohort by diagnosis

|  | **Total** | **POTS*^a^* (including those with aortic root dilation)** | **Percent** |
| --- | --- | --- | --- |
| No hEDS or HSD | 101 | 2 | 2.0% |
| hEDS | 148 | 13 | 8.8% |
| HSD | 333 | 17 | 5.1% |

***^a^*** Abbreviations: hEDS, hypermobile Ehlers-Danlos syndrome; HSD, hypermobility spectrum disorders; POTS, postural orthostatic tachycardia syndrome

**Supplementary Table 2.** Patients with an aortic root z-score >3 self-reported responses regarding their family cardiac history in EDS questionnaire

| Patient | Diagnosis | Aortic Root Z-SCORE | Aneurysm/dissection of aorta in chest cavity (weak artery wall in the chest) | Aneurysm/dissection of abdominal aorta (AA) (weak artery wall in the abdomen) | Brain aneurysm (weak artery wall in the brain) | Heart valve issues |
| --- | --- | --- | --- | --- | --- | --- |
| 1 | hEDS | 4.8 | No | No | Yes | No |
| 2 | hEDS | 3.6 | No | No | No | No |
| 3 | HSD*^a^* | 3.2 | No | Yes | No | No |

*^a^* This patient had the Invitae Connective Tissue Disorder Gene Panel, but results were negative for a variant.

**Supplementary Table 3.** Valve morphology for hEDS and HSD patients (*n* = 568)

| Valve Type*^a^* | Normal | Thickened | Sclerotic |
| --- | --- | --- | --- |
| *hEDS* |  |  |  |
| Aortic Valve | 157 (96.9%) | 2 (1.2%) | 3 (1.9%) |
| Mitral Valve | 155 (97.5%) | 3 (1.9%) | 1 (0.63%) |
| *HSD* |  |  |  |
| Aortic Valve | 361 (97%) | 3 (0.81%) | 8 (2.2%) |
| Mitral Valve | 368 (97.6%) | 7 (1.9%) | 2 (0.53%) |

***^a^*** All rows do not add up to 568 due to missing data for some patients

**Supplementary Table 4.** Mitral valve prolapse patient summary including possible mitral valve prolapse (MVP) (*n* = 15)

| Patient | Sex | Diagnosis*^a^* | Age | MVP Type |
| --- | --- | --- | --- | --- |
| 1 | Female | hEDS | 42 | Possible Anterior Leaflet |
| 2 | Female | HSD | 41 | Bileaflet |
| 3 | Female | hEDS | 38 | Bileaflet |
| 4 | Female | hEDS | 49 | Bileaflet |
| 5 | Female | HSD | 24 | NR |
| 6 | Female | hEDS | 70 | Anterior Leaflet |
| 7 | Female | HSD | 57 | NR |
| 8 | Female | hEDS | 18 | Bileaflet |
| 9 | Female | hEDS | 57 | Bileaflet |
| 10 | Female | hEDS | 23 | Bileaflet |
| 11 | Female | HSD | 31 | Bileaflet |
| 12 | Female | HSD | 34 | Possible Anterior Leaflet |
| 13 | Female | HSD | 52 | Bileaflet |
| 14 | Female | HSD | 46 | Posterior Leaflet |
| 15 | Female | HSD | 31 | Posterior Leaflet |

***^a^*** Abbreviations: hEDS, hypermobile Ehlers-Danlos syndrome; HSD, hypermobility spectrum disorders; MVP, mitral valve prolapse; NR, not reported.

**Supplemental Table 5.** Notable cardiac conditions among all possible mitral valve prolapse patients (*n* = 15)

| Patient | Sex | Diagnosis | Age at ECHO*^a^* | Condition | MVP Type | MVR | MV morphology |
| --- | --- | --- | --- | --- | --- | --- | --- |
| 1 | Female | hEDS | 18 | Atrial Fibrillation | bileaflet | mild | thickened mitral valve |
| 2 | Female | hEDS | 42 | Arrhythmia | possible anterior leaflet | trivial | normal |
| 3 | Female | HSD | 24 | Arrhythmia | NR | trivial | NR |
| 4 | Female | HSD | 46 | Arrhythmia | posterior leaflet | mild | mildly thickened mitral valve |

***^a^*** Abbreviations: hEDS, hypermobile Ehlers-Danlos syndrome; HSD, hypermobility spectrum disorder; ECHO, echocardiogram; MVR, mitral valve regurgitation; NR, not reported; MV, mitral valve; MVP, mitral valve prolapse.

**Supplemental Table 6.** Mitral valve prolapse severity measurements for all possible mitral valve prolapse patients (*n* = 15)

| Patient | Age at ECHO*^a^* | MVR | MV Deceleration Time (s) | MV E-Wave Peak Velocity (m/s) | MV A-Wave Peak Velocity (m/s) | MV E to A Ratio at Baseline | MVR ERO; VOLUME (PISA) |
| --- | --- | --- | --- | --- | --- | --- | --- |
| 1 | 42 | Trivial | 308 | 0.4 | 0.4 | 1 | NR |
| 2 | 41 | Trivial | 198 | 0.7 | 0.4 | 1.75 | NR |
| 3 | 38 | Trivial | 355 | 0.7 | 0.9 | 0.78 | NR |
| 4 | 49 | Trivial | 148 | 0.9 | 0.7 | 1.29 | NR |
| 5 | 24 | Trivial | 130 | 0.7 | 0.7 | 1 | NR |
| 6 | 70 | Moderate | NR | 1.2 | 1.4 | 0.86 | 0.3; 68. |
| 7 | 57 | Mild | 152 | 0.6 | 0.4 | 1.5 | NR |
| 8 | 18 | Mild | 193 | 1.1 | 0.5 | 2.2 | NR |
| 9 | 57 | Mild-Moderate | 254 | 0.7 | 0.6 | 1.17 | 0.2; 39 |
| 10 | 23 | Mild | NR | NR | NR | NR | 0.1; 9 |
| 11 | 31 | Trivial | 224 | 0.6 | 0.5 | 1.2 | NR |
| 12 | 34 | Trivial | 165 | 0.5 | 0.6 | 0.83 | NR |
| 13 | 52 | Mild | 184 | 0.6 | 0.4 | 1.5 | NR |
| 14 | 46 | Mild | 164 | 0.7 | 0.4 | 1.75 | NR |
| 15 | 31 | Trivial | 173 | 0.9 | 0.4 | 2.25 | NR |

***^a^*** Abbreviations: ECHO, echocardiogram; MVR, mitral valve regurgitation; NR, not reported; MV, mitral valve; ERO, effective regurgitant orifice; PISA, Proximal Isovelocity Surface Area

**Supplementary Table 7.** Normalized mitral valve prolapse severity measurements for all possible mitral valve prolapse patients (*n* = 15)

| Patient | *^a^* MV Deceleration Time Z-SCORE | MV E-Wave Peak Velocity Z-SCORE | MV A-Wave Peak Velocity Z-SCORE | MV E to A Ratio Z-SCORE |
| --- | --- | --- | --- | --- |
| 1 | 3.05 | -2.18 | -1.67 | -0.94 |
| 2 | -0.20 | -0.41 | -1.67 | 1.33 |
| 3 | 4.84 | -0.73 | 1.72 | -2.00 |
| 4 | -1.67 | 0.76 | 0.83 | -0.06 |
| 5 | -1.80 | -0.88 | 2.27 | -1.73 |
| 6 | N/A | 4.00 | 2.94 | 1.27 |
| 7 | -1.53 | -0.63 | -2.07 | 1.84 |
| 8 | 0.19 | 0.00 | 0.33 | -0.48 |
| 9 | 1.13 | 0.00 | -0.64 | 0.52 |
| 10 | N/A | N/A | N/A | N/A |
| 11 | 0.84 | -1.18 | -0.50 | -0.86 |
| 12 | -0.97 | -1.64 | 0.06 | -1.86 |
| 13 | -0.70 | -0.63 | -2.07 | 1.84 |
| 14 | -1.20 | -0.41 | -1.67 | 1.33 |
| 15 | -0.72 | 0.18 | -1.06 | 1.97 |

***^a^*** Abbreviations: MV, mitral valve

**Supplementary Table 8.** Gene panel summary*^a^* for hEDS and HSD patients who tested positive for a genetic variant (*n* = 16)

| Age | Sex | hEDS  HSD | CV issues*^b^* | Test | Gene | Zygosity | cDNA change | AA change | Classification | Clinical genomics consult |
| --- | --- | --- | --- | --- | --- | --- | --- | --- | --- | --- |
| 47 | F | HSD | None | EDS gene panel | *ADAMTS2* | Het | c.3195C>T | p.Gly1065= | VUS | Enzyme needed for collagen synthesis. Recessive- mutations anticipated on both alleles. Associated with dermatosparaxis EDS (dEDS) (EDS type VIIC). If pathogenic, could be a carrier. |
| 28 | F | hEDS | None | EDS gene panel | *ATP7A* | Het | c.2519A>T | p.Glu840Val | VUS | X chromosome. Copper shuttle important in all tissues including connective tissue. Pathogenic variants can cause X-linked Menkes syndrome and the milder occipital horn syndrome (also known as X-linked cutis laxa). |
|  |  |  |  |  | *COL1A1* | Het | c.1717G>C | p.Ala573Pro | VUS | Type I collagen found in connective tissue. If pathogenic, associated with osteogenesis imperfecta (types I-IV), Caffey disease, or EDS arthrochalasia type 1. |
| 28 | F | HSD | Mild pulmonary valve regurgitation. | EDS gene panel | *COL1A2* | Het | c.1475T>C | p.Ile492Thr | VUS | One of the chains for Type I collagen in connective tissue. If pathogenic, associated with Stickler syndrome. |
| 31 | F | HSD | None | EDS gene panel | *COL5A1* | Het | c.3023C>T | p.Thr1008Met | VUS | Low abundance collagen. Mutations associated with Type I and II EDS, typically associated joint hypermobility, hyper elastic/ stretchable skin, recurrent injuries, and atrophic scars. |
| 32 | F | HSD | None | EDS gene panel | *COL5A1* | Het | c.3812C>T | p.Pro1271Leu | VUS | Low abundance collagen. Mutations associated with Type I and II EDS, typically associated joint hypermobility, hyper elastic/ stretchable skin, recurrent injuries, and atrophic scars. |
| 37 | F | HSD | None | OSM | *Factor 5 Leiden* | Het | NR | NR | VUS | Mutated form of Factor V which increases clotting. Heterozygous for Factor V mutation which on its own is not an indication for long-term anticoagulation therapy. |
| 24 | F | HSD | Mild pulmonary valve regurgitation | OSM | *FBN1* | Het | c.98A>G | p.Asn33Ser | VUS | Associated with autosomal dominant Marfan syndrome, Mass syndrome, thoracic aortic aneurysm, and aortic dissection. |
| 22 | F | HSD | None | EDS gene panel | *FLNA* | Het | c.5686G>A | p.Gly1896Ser | VUS | Filamin A interacts with actin for the cytoskeleton. X-linked. Mutation can lead to periventricular nodular heterotopia, and other brain malformations, and occasionally also findings of aortic aneurysms. Otopalatodigital syndrome, type I in females. |
| 49 | F | hEDS | Mild aortic regurgitation. | Marfan and related panel, B | *FLNA* | Het | c.6151G>A | p.Glu2051Lys | VUS | Filamin A interacts with actin for the cytoskeleton. X-linked. Mutation can lead to periventricular nodular heterotopia, and other brain malformations, and occasionally also findings of aortic aneurysms. Otopalatodigital syndrome, type I in females. |
| 72 | F | hEDS | Mitral valve anterior leaflet prolapse. Moderate mitral valve regurgitation. Mild aortic and pulmonary regurgitation. | EDS gene panel | *FLNA* | Het | c.1580G>A | p.Arg527His | VUS | Filamin A interacts with actin for the cytoskeleton. X-linked. Mutation can lead to periventricular nodular heterotopia, and other brain malformations, and occasionally also findings of aortic aneurysms. Otopalatodigital syndrome, type I in females. |
| 42 | F | HSD | None | Hemochromatosis HFE gene analysis, B | *HFE C282Y* | Het | NR | NR | VUS | A carrier of hereditary hemochromatosis (HH). Most commonly associated with C282Y variant. Can lead to joint pain and heart failure. |
| 52 | F | HSD | None | Hemochromatosis HFE gene analysis, B | *HFE C282Y* | Het | NR | NR | VUS | A carrier of hereditary hemochromatosis (HH). Most commonly associated with C282Y variant. Can lead to joint pain and heart failure. |
|  |  |  |  |  | *LTBP1* | Het | c.2028 G>C | p.E676D | VUS | Latent TGFb binding protein-1. Not known to be pathogenic, but if pathogenic, only a carrier state. |
| 36 | F | HSD | None | OSM | *MYH7* | Het | NR | NR | VUS | Myosin heavy chain mutations associated with autosomal dominant hypertrophic cardiomyopathy (HCM). |
| 19 | F | hEDS | Bileaflet mitral valve prolapse and thickened mitral valve. Mild aortic, tricuspid, and mitral regurgitation. | EDS gene panel | *PLOD1* | Het | c.1862C>T | p.Ala621Val | VUS | Lysl hydrolase- If this variant were pathogenic, patient would be a carrier. Biallelic pathogenic variants in PLOD1 are associated with autosomal recessive EDS, kyphoscoliotic type. |
| 36 | F | HSD | Mild pulmonary and tricuspid valve regurgitation. | EDS gene panel | *PLOD1* | Het | c.1828C>T | p.Arg610Trp | VUS | Lysl hydrolase- If this variant were pathogenic, patient would be a carrier. Biallelic pathogenic variants in PLOD1 are associated with autosomal recessive EDS, kyphoscoliotic type. |
| 28 | F | HSD | None | Comprehensive arrhythmia sequencing and deletion/duplication panel | *TRDN* | Het | c.1367A>G | Gln456Arg | VUS | Triadin gene regulates calcium-induced muscle contraction. |

***^a^*** Patients are listed alphabetically according to gene variant. *^b^* Abbreviations: AA, amino acid; ADAMTS2, disintegrin and metalloproteinase with thrombospondin motifs 2; CV, cardiovascular; FBN1, fibrillin-1; FLNA, filamin A; hEDS, hypermobile Ehlers-Danlos syndrome; Het, heterozygous; HFE, human homeostatic iron regulator protein; HSD, hypermobility spectrum disorders; NR, not reported; TGF, transforming growth factor; VUS, variant of uncertain significance.
